# Reprogramming, Circular Reasoning and Self versus Non-self: One-Stop Shopping with RNA Editing

**DOI:** 10.3389/fgene.2016.00100

**Published:** 2016-06-07

**Authors:** Yiannis A. Savva, Ali Rezaei, Georges St. Laurent, Robert A. Reenan

**Affiliations:** Department of Molecular Biology, Cell Biology and Biochemistry, Brown University, ProvidenceRI, USA

**Keywords:** RNA editing, RNA metabolism, RNA splicing, RNA silencing, circular RNAs, immune responses, neurological disorders, autoimmune disease

## Abstract

Transcription of genetic information from archival DNA into RNA molecule working copies is vital for proper cellular function and is highly accurate. In turn, RNAs serve structural, enzymatic, and regulatory roles, as well as being informational templates for the ribosomal translation of proteins. Following RNA synthesis, maturing of RNA molecules occurs through various RNA processing events. One component of the collection of processes involving RNA species, broadly defined as RNA metabolism, is the RNA-editing pathway and is found in all animals. Acting specifically on RNA substrates with double-stranded character, RNA editing has been shown to regulate a plethora of genomic outputs, including gene recoding, RNA splicing, biogenesis and targeting actions of microRNAs and small interfering RNAs, and global gene expression. Recent evidence suggests that RNA modifications mediated via RNA editing influence the biogenesis of circular RNAs and safeguard against aberrant innate immune responses generated to endogenous RNA sources. These novel roles have the potential to contribute new insights into molecular mechanisms underlying pathogenesis mediated by mishandling of double-stranded RNA. Here, we discuss recent advances in the field, which highlight novel roles associated with the RNA-editing process and emphasize their importance during cellular RNA metabolism. In addition, we highlight the relevance of these newly discovered roles in the context of neurological disorders and the more general concept of innate recognition of self versus non-self.

## Introduction

The most prevalent type of RNA editing in metazoans is the adenosine to inosine (A-to-I) modification, mediated by a highly conserved protein family known as adenosine deaminases acting on RNA (ADAR; Savva et al., 2012b). Acting on RNAs with substantial degrees of double-strandedness (dsRNAs), ADAR enzymes have the capacity to bind and modify specific adenosines to inosines in short and imperfect dsRNA substrates (Nishikura, 2010). Conversely, in long and perfectly base-paired dsRNA molecules, ADARs exhibit a promiscuous catalytic activity that modifies up to 40–50% of adenosines, a phenomenon referred to as hyper-editing (Bass, 2002). Inosine nucleosides in RNA are interpreted as guanosines by the cellular machineries involved in RNA metabolism, including polymerases, the spliceosome and ribosome (Basilio et al., 1962). Thus, ADAR-mediated modifications in RNA molecules inherently alter RNA metabolism on multiple levels, generating an increased variety of transcriptional outputs that enhance eukaryotic molecular complexity and serve as a source of variation for the generation of evolutionary novelty. Characterization of RNA-editing landscapes from a broad range of phyla, using next-generation sequencing technologies, suggests that ADAR modifications are more widespread than previously thought and are distributed throughout genomes in highly species-specific patterns. Despite variations between RNA-editing landscapes across model organisms, editing sites are observed in both coding and non-coding regions of the genome, with the latter being the most prevalent (St Laurent et al., 2013; Zhao et al., 2015). Similarly, RNA-editing sites in humans are over-represented in non-coding *Alu* repeats (Ramaswami et al., 2012; Bazak et al., 2014), the most abundant transposable element existing in the genome, corresponding to approximately 10% of its content (Lander et al., 2001).

RNA-editing enzymes are enriched in the nuclear compartment and expressed predominantly within the nervous system (Savva et al., 2012b). This specific localization pattern suggests a pivotal role of these enzymes for proper nervous system function. Indeed, the primary function of ADARs is thought to be in preserving nervous system integrity, as exemplified by neurological phenotypes of RNA-editing-deficient genetic models. Specifically, invertebrates lacking all ADAR activity exhibit severe neurological defects and behavioral abnormalities. For example, loss of function of the single *Adar* in *Drosophila* leads to frequent seizures, chronic uncoordination, and age-dependent neurodegeneration (Palladino et al., 2000). Furthermore, *Caenorhabditis elegans* (*C. elegans*) lacking RNA-editing activity through the deletion of both encoded *adar* genes exhibit defects in chemotaxis (Tonkin et al., 2002). In contrast to ADAR deficiencies in invertebrates, mice lacking either ADAR1 or ADAR2 editing enzymes result in lethal phenotypes. Deletion of ADAR1 leads to embryonic lethality accompanied by elevated cellular apoptosis (Wang et al., 2004), while mice lacking ADAR2 exhibit severe seizure episodes characteristic of juvenile-onset epilepsy that result in lethality early in life (Higuchi et al., 2000). Two observations suggest that RNA-editing systems are functionally pleiotropic in regulating distinct physiologically essential RNA pathways. First, the chemotaxis defect exhibited by *adar* null *C. elegans* is rescued by mutants impaired in RNA interference (RNAi) response (Tonkin and Bass, 2003). This observation suggests that without RNA editing, improper dsRNAs enter the RNAi pathway and trigger spurious silencing responses. Such a result is confirmed by the analysis of small RNAs generated in these *adar* null animals. In this study, the absence of *adar* caused the appearance of new classes of small interfering RNAs (siRNAs) from what the authors named, *adar*-dependent loci (ADLs; Wu et al., 2011). Second, the lethality observed in ADAR2 null mice can be rescued by the edited version of the glutamate receptor GluR2 (Higuchi et al., 2000). Therefore, depending upon species context, some of the numerous fates of edited RNA molecules are more physiologically relevant compared to others operating within the same transcriptome.

## General Fates of Edited RNAs

### Genomic Recoding

**FIGURE 1:**
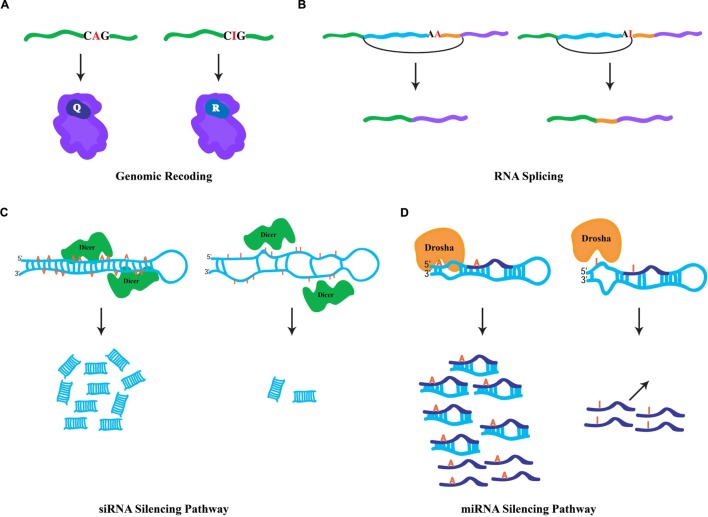
**General fates of edited RNAs.** **(A)** Specific RNA editing in coding region of a pre-mRNA. ADAR-mediated hydrolytic deamination of the adenine base within the glutamic acid codon (Q) is interpreted by the ribosomal machinery as an arginine codon (R), leading to an amino acid substitution. **(B)** Specific RNA editing in non-coding regions can generate novel 3′ splice acceptor sites (AA → AG). In mammals, the ADAR2 RNA-editing enzyme modifies its own transcript to generate a novel splicing signal that results in the inclusion of 47 nucleotides in the mature RNA. **(C)** Hyper-editing of dsRNA molecules leads to inefficient Dicer processing. Hyper-editing antagonizes RNA-mediated silencing responses through the generation of fewer siRNAs. **(D)** Specific RNA editing near the base of miRNA precursors leads in the inhibition of Drosha cleavage, which prevents the processing of mature miRNAs. In addition, specific RNA editing within the “seed” region of the mature miRNA may result in redirection to a new target.

Since ribosomes interpret inosines as guanosines, the capacity to generate protein products that are not literally encoded by genomic DNA is enabled by RNA editing in coding regions. Also known as genomic recoding, this phenomenon extends the genetic information potential through diversification of the protein repertoire, analogous to alternative splicing (Nilsen and Graveley, 2010). In the nucleus and during the synthesis of nascent transcripts, ADAR enzymes bind to dsRNA structures generally formed by highly conserved intronic sequences, which are complementary with the exon to be edited. Occurring co-transcriptionally (Rodriguez et al., 2012), the short and imperfectly base-paired nature of the dsRNA substrates allows ADARs to choose specific adenosines for modification within exonic sequences (Bass, 2002), directed by loops and bulges in the dsRNA structure. Edited RNA templates are subsequently exported to the cytoplasm and translated by the ribosomal machinery. For instance, a specific RNA-editing event within a glutamic acid codon (CAG(Q) → C**I**G) is interpreted by the ribosome as CGG(R) (**Figure 1A**), and results in the insertion of the charged residue arginine in the polypeptide chain rather than the encoded polar residue glutamine. Genomic recoding is extensively utilized in *Drosophila* as a means of neuronal proteome diversification (St Laurent et al., 2013), while in vertebrates this kind of RNA editing seems to be limited (Lagarrigue et al., 2013). Intriguingly, proteins involved in neurotransmission are the major targets of specific editing (Hoopengardner et al., 2003). However, recent studies suggest that RNA editing can additionally target transcripts encoding proteins involved in a variety of cellular functions including transcription, RNA splicing, protein metabolism, and DNA replication (Graveley et al., 2011; St Laurent et al., 2013). Generally, genomic recoding events can influence protein function, and in some cases this fine-tuning effect can have broad cellular consequences. For example, a specific RNA-editing event that leads to a quite conservative missense amino acid substitution dramatically alters the rate of inactivation in human potassium (K^+^) channels (Bhalla et al., 2004), while a single RNA-editing site within the *Adar* transcript in *Drosophila* reshapes the global landscape of editing events in a manner that impacts complex adult behaviors (Savva et al., 2012a) and has even more dramatic consequences for heterochromatic gene silencing (Savva et al., 2013).

### RNA Splicing

Since RNA editing occurs cotranscriptionally, specific ADAR modifications can influence downstream RNA processing events. Given that the majority of editing sites are found within intronic sequences, editing has the capacity to influence RNA splicing. More specifically, due to the canonical nature of 5′ splice donor sites (GU) and 3′ splice acceptor sites (AG), specific RNA editing in introns can generate novel splicing signals (e.g., AU-to-**I**U → GU (donor) or AA-to-A**I** → AG(acceptor); Nishikura, 2010). For example, RNA editing in an intronic *Alu* element within the human nuclear prelamin A recognition factor generates a novel 3′ splicing acceptor site regulating its exonization in a tissue-specific pattern (Lev-Maor et al., 2007). Similarly, the mammalian ADAR2-editing enzyme generates a new acceptor site in its own transcript that results in a 47 nucleotide inclusion (**Figure 1B**) and subsequent generation of a hypomorphic allele (Rueter et al., 1999), which leads to reduction in RNA-editing levels at multiple adenosine targets (Feng et al., 2006). Correspondingly, RNA editing in a 3′ acceptor splice site (AG-to-**I**G → GG) can prevent its recognition by the spliceosome machinery. The observation that ADAR1 knockdown leads to aberrant exonization of an *Alu* element in the seryl-tRNA synthetase transcript, and suggests that RNA editing also plays a destructive role in eliminating unwarranted acceptor sites to ensure proper splicing (Sakurai et al., 2010).

### RNA Silencing

Cellular defense against endogenous and exogenous parasitic nucleic acids (such as transposons and viruses, respectively) is achieved by dsRNA molecules, which trigger a highly conserved biological response known as RNAi (Hannon, 2002). In addition to its safeguarding roles, RNAi also regulates gene expression through target transcript cleavage or by translation repression. Central to RNAi-mediated gene regulation are two kinds of small RNA species, siRNAs and microRNAs (miRNAs; Ghildiyal and Zamore, 2009). These small RNA species are generated via Dicer processing of dsRNA triggers (Bernstein et al., 2001), whose formation is mediated by endogenous genomic sequences with near-perfect base complentarity. For the biogenesis of siRNAs, Dicer enzymes process long and perfectly base-paired dsRNA molecules usually formed by transposable element sequences (Kawamura et al., 2008). Such dsRNA sources can also serve as ADAR substrates. Similarly, RNA-editing enzymes are capable of binding the same substrates as Dicer, hyper-editing the dsRNA molecules and destroying their near-perfect duplex nature and leading to inefficient Dicer processing (**Figure 1C**; Nishikura, 2006). Thus, hyper-editing has the capacity to antagonize cellular RNAi responses through interfering with siRNA biogenesis (Scadden and Smith, 2001). This assertion is borne out by the observation that a significant fraction of siRNA effector molecules found in AGO2 RISC complexes contain a single A-to-G mismatch, implying the action of ADAR on siRNA precursors does not completely prevent their appearance in silencing complexes (Kawamura et al., 2008). Intriguingly, ADAR activity can regulate heterochromatin formation by associating with dsRNA sources produced from transposable elements, altering gene expression of neighboring genomic regions (Savva et al., 2013). Contrary to the siRNA pathway, the dsRNA triggers for miRNA biogenesis are shorter and usually contain bulges and loops, resulting in specific editing (Luciano et al., 2004; Blow et al., 2006). Moreover, specific editing regulates the miRNA pathway at numerous levels (**Figure 1D**). First, as miRNA precursors are edited, pre-miRNA cleavage and other processing steps of the pathway can be inhibited (Yang et al., 2006). Second, the targeting step, which requires complementarity between a miRNA and its target sequence, may potentially reroute the miRNA target site recognition to new mRNAs due to specific editing within the miRNA “seed” region. Indeed, a single RNA-editing event is sufficient to redirect a miRNA to a new complementary target (Kawahara et al., 2007). Finally, the observation that ADAR modifications are abundant at miRNA target sites suggests that RNA editing can regulate gene expression by destruction of miRNA/target complementarity (Gu et al., 2012).

## RNA Editing and Circular RNAs

**FIGURE 2:**
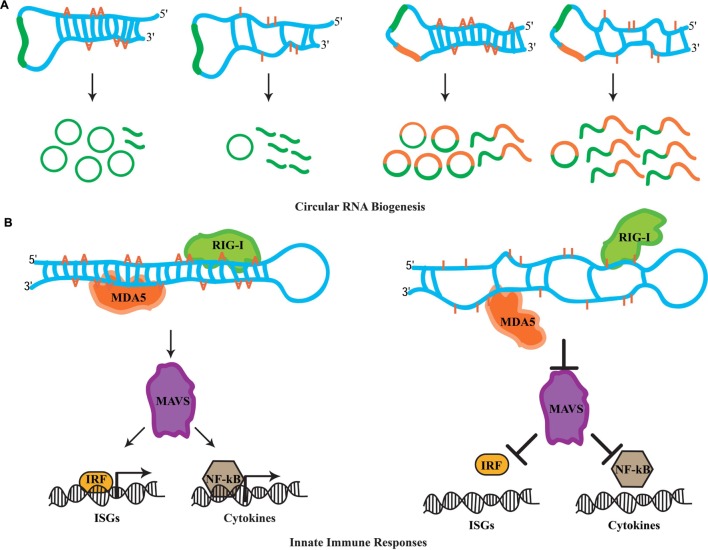
**Novel roles for RNA editing.** **(A)** The biogenesis of circular RNAs involves the formation of dsRNA duplexes mediated by intronic sequences flanking the exon(s) destined for circularization. These dsRNA molecules can serve as ADAR substrates. It is currently thought that hyper-editing destroys the dsRNA nature of such substrates, which promotes linear splicing and restricts exon circularization. Examples of how RNA editing antagonizes the biogenesis of circRNAs formed by single exon and two exons are depicted. **(B)** In the cytoplasm, RIG-I and MDA5 act as sensors for dsRNA molecules. Upon recognition, these sensors activate MAVS, which triggers the innate immune response. MAVS-mediated signals result in the translocation of interferon-regulatory factors (IRFs) and NF-κB into the nucleus, which initiates the transcription of ISG and cytokine genes. Hyper-editing of immunoreactive dsRNAs prevents detection by RIG-I and MDA5, inhibiting aberrant innate immune responses toward endogenous dsRNA sources.

Transcriptional profiling studies in metazoans have revealed mysterious new RNA species comprising unbroken circles (Memczak et al., 2013; Jeck and Sharpless, 2014; Lasda and Parker, 2014). Termed circular RNAs (circRNAs), these RNA species are generated through a non-linear splicing mechanism in which a canonical 5′ donor site of an exon is spliced to an upstream 3′ acceptor splice site (Ashwal-Fluss et al., 2014; Starke et al., 2015). While in humans circRNAs can be detected in diverse cell types (Salzman et al., 2012), recent evidence suggests that they are highly enriched in the nervous system, specifically at synapses (Westholm et al., 2014; Rybak-Wolf et al., 2015). A hallmark for circRNA biogenesis in mammals is the presence of complementarity between inverted intronic sequences that flank the exon destined for circularization. Mechanistically, these sequences can base pair extensively forming stem-loop dsRNA molecules, wherein the exon(s) to be circularized are sequestered within the loop sequences. *Alu* repetitive sequences are highly associated with exon circularization (Jeck et al., 2013; Zhang et al., 2014). Strikingly, A-to-I substitutions in introns flanking splice sites of circularization are highly enriched (Ivanov et al., 2015). Moreover, ADAR knockdown leads to accumulation of circRNAs in human cells (Ivanov et al., 2015). Similarly, several mouse circRNAs are upregulated upon decreasing ADAR expression (Rybak-Wolf et al., 2015). This antagonistic effect between RNA-editing enzymes and circRNA biogenesis is a conserved feature in invertebrates. First, intronic sequences flanking circRNAs are enriched for hyper-editing events in *C. elegans* (Ivanov et al., 2015). Second, *Drosophila* raised at 29°C exhibit elevated levels of circRNAs when compared to flies maintained at 18°C (Rybak-Wolf et al., 2015). The accumulation of circRNAs at higher temperatures is thought to occur due to a decrease in ADAR expression at these temperatures, as a recent study shows elevated temperature dynamically downregulates levels of the ADAR protein (Rieder et al., 2015). Clearly, circRNA biogenesis is highly regulated through ADAR activity; however how this is achieved mechanistically is not yet clear. While the biological roles of circRNAs are currently unknown, evidence suggests that circRNAs can act as sponges for the assembly of miRNAs and accumulate during the aging of the nervous system (Hansen et al., 2013; Westholm et al., 2014). Nevertheless, whatever the functions of these circRNAs might be, RNA editing has the capacity to fine-tune downstream biological phenomena by antagonizing the biogenesis of circRNAs (**Figure 2A**).

## RNA Editing and Innate Immune Responses to dsRNAs

Cellular infection by viruses activates a protective mechanism that involves an inflammatory response (Medzhitov, 2008). The physiological role of this inflamatory response is to provide necessary stimuli required by the host to establish a potent defense mechanism. Referred to as the innate immune response, it is triggered by bacteria and viruses without the need for adaptive immunity. In particular, the recognition of foreign dsRNAs generated during the initial cycles of viral replication is a potent inducer of innate immune pathways (Akira et al., 2006). In vertebrates, the retinoic acid-inducible gene I (RIG-I) and the melanoma differentiation-associated gene 5 (MDA5) operate as sensors for the recognition of foreign dsRNA molecules (Takeuchi and Akira, 2010). In the cytoplasm, RIG-I recognizes dsRNAs that are up to 1 kb in legth, while MAD5 senses longer RNA duplexes. Despite this discriminatory recognition, both sensors trigger the actions of a mitochondrial antiviral-signaling protein, MAVS, which signals for the activation of a cascade of events. This cascade involves several factors that orchestrate the expression of cytokines and type I interferons (IFNs) genes required for multiple defense responses (Takeuchi and Akira, 2010). It has been recently realized that genomes are transcribed in their entireties through pervasive transcription (Carninci et al., 2005; Djebali et al., 2012), generating a myriad of RNA species that participate in diverse cellular functions. Moreover, the identification of endogenous RNA-based silencing pathways suggests that dsRNA molecules are generated by a variety of genomic sources (Nilsen, 2008). Due to their nature, endogenously generated dsRNA duplexes are highly structurally similar to those generated through viral replication after infection. This raises the question of how cells are able to discriminate endogenous dsRNA molecules from exogenous and therefore avoid aberrant immune responces. Previous observations suggest that endogenous RNAs marked with specific nucleoside modifications avoid detection by sensor proteins of the immune response (Kariko et al., 2005). Intriguingly, synthetic dsRNAs containing multiple IU pairs, which mimic the hyper-editing activity of RNA-editing enzymes, fail to induce innate immune responses (Vitali and Scadden, 2010). Thus, it was proposed that the presence of IU pairs in dsRNA duplexes interfere with the detection process mediated by the RIG-I and MAD5 sensors, suggesting that RNA editing in principal can regulate innate immune responses. Indeed, two recent studies have uncovered that RNA editing regulates the cascade of events that lead to the activation of innate immune responses. Moreover, this pathway acts upstream to hyper-edit naturally occuring (self) dsRNA duplexes in order to avoid detection as foreign dsRNA (non-self) that otherwise would elicit abberant immune responses.

In these studies, it was reasoned that the elevated cellular apoptosis exhibited by ADAR1 null mice is due to induction of immune/inflammatory responses triggered by non-edited, endogenous dsRNA sources. In agreement with this notion, Mannion et al. (2014) examined the transcriptional profiling in ADAR1 null embryos and observed that transcripts of interferon-stimulated genes (ISGs) were elevated significantly. Moreover, the Adar null (*Adar1*^(-/-)^) mouse embryonic lethal phenotype was substantially rescued to pup birth in *Adar1*^(-/-)^/*Mavs*^(-/-)^ double-mutant mice. Finally, transfection of dsRNA species containing multiple IU pairs was unable to elicit an immune response in fibroblast cells of ADAR1-deficient mice, which indicates that editing activity is necessary for blocking the response. In a similar study, Liddicoat et al. (2015) generated an ADAR1 editing-activity-deficient mouse allele (*Adar1^E861A/E861A^*). Through transcriptional profiling of homozygous mutant mice, they observed an atypical upregulation of ISGs, highly similar to the one observed in the ADAR1 protein null mice (Liddicoat et al., 2015). Furthermore, through analysis of the ADAR1 editing landscape they revealed that the major substrates of this RNA-editing ezyme are dsRNA duplexes formed by 3′ UTR sequences. They reasoned that the absence of IU pairs from these dsRNAs can elicit aberrant immune responces through MDA5 sensing. Indeed, generating *Adar1^E861A/E861A^*/MDA5^(-/-)^ double-mutant mice also rescued the embryonic lethality phenotype suggesting that MDA5 is the principal sensor of non-edited endogenous dsRNAs. Clearly, these observations suggest a pivotal role for RNA editing in the regulation of innate immune responses toward self-generated dsRNA molecules. More importantly, ADAR1 acts hierarchically upstream in the immune cascade and through RNA editing it fingerprints dsRNAs as endogenous, thus preventing undesired cellular immune responses and allowing robust measures to be taken against invading non-self dsRNA signaling events (**Figure 2B**).

## Conclusion

A universal property of RNA, and trait of transcriptomes in general, is the generation of RNA molecules with varying degrees of double-strandedness that orchestrate diverse cellular functions. Despite variation in length, many endogenous molecules with dsRNA character intersect with the A-to-I RNA-editing system, since no sequence specificity is required for ADAR binding and editing (Nishikura et al., 1991).

Rather, ADAR modification conceptually generates two radically opposed forces determined by substrate structure. When structures are imperfect dsRNA, only specific editing events occur. Because, in general, these imperfect structures involve conserved secondary and tertiary RNA interactions of exquisite detail, specific editing must be seen as a creative force in the evolutionary tool-kit – a novelty generator. Conversely, less specific hyper-editing of near-perfect duplexes serves a preventative or intervening role, competing with other enzymes that have different designs on the fate of long perfect dsRNA. Deconvolution of these opposing roles of editing in organismal phenotype will remain a challenge into the near future.

RNA-editing enzymes have the inherent capacity to regulate many dinstinct pathways that are involved in RNA metabolism and serve to fine-tune and optimize transcriptional outputs (Bahn et al., 2015). Although, anomalies in RNA editing are associated with multiple neurological disorders such as epilepsy, schizophrenia, and amyotrophic lateral sclerosis (ALS; Slotkin and Nishikura, 2013), the molecular determinants of ADAR-mediated pathogenesis still remain elusive. In principle, mutations that affect editing activity (Crow et al., 2015) or lead to abnormal ADAR sequestration (Donnelly et al., 2013) can both lead to disease phenotypes due to detrimental impacts imposed on the known general fates of edited RNAs. In addition, as these recent studies suggest, a severe reduction in RNA editing may lead to the abnormal accumulation of circular RNAs and elicit aberrant innate immune responses. Future research should aim to understand how these novel RNA-editing roles may contribute to the etiology of neurological disorders.

Previously, multiple missense mutations in the *Adar1* gene were identified in individuals diagnosed with AicardiGoutières syndrome (AGS), an autoimmune disorder (Rice et al., 2012). In addition, individuals with AGS-associated *Adar1* mutations exhibited high levels of expression for a number of IFN-stimulated genes, a phenotype observed previously in ADAR1-deficient mice (Hartner et al., 2009). Therefore, it was proposed that ADAR1 theoretically could suppress immunoreactive dsRNA molecules through hyper-editing and control aberrant immune reponses. Certainly, the new role for the A-to-I RNA-editing system provided by Mannion et al. (2014) and Liddicoat et al. (2015) contributed significantly to a better understanding of how *Adar1* mutations can result in autoimmune diseases. Thus, it is becoming increasingly clear that ADAR-mediated modifications occupy a central role in cellular physiology. Therefore, a more comprehensive understanding on how RNA-editing systems regulate global cellular RNA metabolism can shed light in the development of therapeutic strategies of ADAR-mediated human diseases by providing insights into their pathogenesis.

## Author Contributions

All authors listed, have made substantial, direct and intellectual contribution to the work, and approved it for publication.

## Conflict of Interest Statement

The authors declare that the research was conducted in the absence of any commercial or financial relationships that could be construed as a potential conflict of interest.
